# Proteome-Wide Analysis of Functional Divergence in Bacteria: Exploring a Host of Ecological Adaptations

**DOI:** 10.1371/journal.pone.0035659

**Published:** 2012-04-26

**Authors:** Brian E. Caffrey, Tom A. Williams, Xiaowei Jiang, Christina Toft, Karsten Hokamp, Mario A. Fares

**Affiliations:** 1 Department of Genetics, University of Dublin, Trinity College, Dublin, Ireland; 2 Department of Molecular Evolution, Evolutionary Biology Centre, Uppsala University, Uppsala, Sweden; 3 Integrative Systems Biology Group, Instituto de Biología Molecular y Celular de Plantas, CSIC-Universidad Politécnica de Valencia (UPV), Valencia, Spain; J. Craig Venter Institute, United States of America

## Abstract

Functional divergence is the process by which new genes and functions originate through the modification of existing ones. Both genetic and environmental factors influence the evolution of new functions, including gene duplication or changes in the ecological requirements of an organism. Novel functions emerge at the expense of ancestral ones and are generally accompanied by changes in the selective forces at constrained protein regions. We present software capable of analyzing whole proteomes, identifying putative amino acid replacements leading to functional change in each protein and performing statistical tests on all tabulated data. We apply this method to 750 complete bacterial proteomes to identify high-level patterns of functional divergence and link these patterns to ecological adaptations. Proteome-wide analyses of functional divergence in bacteria with different ecologies reveal a separation between proteins involved in information processing (Ribosome biogenesis etc.) and those which are dependent on the environment (energy metabolism, defense etc.). We show that the evolution of pathogenic and symbiotic bacteria is constrained by their association with the host, and also identify unusual events of functional divergence even in well-studied bacteria such as *Escherichia coli*. We present a description of the roles of phylogeny and ecology in functional divergence at the level of entire proteomes in bacteria.

## Introduction

Most new genes, functions, and activities originate through the modification of existing ones. The evolutionary process that gives rise to functional differences between related genes is called functional divergence [1], [2]. At the species level, functional diversification is primarily associated with adaptive radiations, when a single ancestor differentiates into multiple descendant species, each adapting by natural selection to one of a new set of ecological niches (Schluter 2000) [3]. Following this theory, environmental variation triggers divergent natural selection, leading to the emergence of niche specialists. In many cases, species under the same ecological conditions differ in their ability to adapt to new niches, even when they stem from the same ancestor [4], [5]. Therefore, other factors such as genetic constraints also play an important role in the process of functional divergence.

The process of functional divergence, or departure of a gene from its ancestral function, is constrained by the requirement to maintain the original function: mutations that confer a new function are likely to interfere with the ancestral function and therefore are eliminated by negative selection. This constraint can be relaxed when selection for the ancestral function is weakened, either through gene duplication (and therefore redundancy), or through changes to the environment inhabited by the organism or a combination of both these factors. After gene duplication, one copy of the gene can be free to evolve in a new direction if the other continues to perform the ancestral function (neofunctionalization). Alternatively, ancestral functions can be partitioned between the two gene copies, potentially leading to later specialization or subfunctionalization [1], [2], [6], [7], [8]. Major changes in the environment or ecological niche can also lead to a relaxation of selective constraints on ancestral functions, although this process is less well characterized. For example, endosymbiotic bacteria have lost many of the genes their free-living relatives need to obtain nutrients from the environment [9], but have also experienced functional divergence in certain genes [10], [11].

Prokaryotes are extraordinarily rich in biological diversity, whether measured in terms of number of species [12], [13], habitat range [14], or the breadth of energy sources and biochemical pathways they can exploit in order to survive [15]. Even photosynthesis and oxidative phosphorylation – the mainstays of eukaryotic energy metabolism – are bacterial inventions acquired by endosymbiosis during early eukaryote evolution [16]. How did this prokaryotic diversity evolve, particularly when the fixation of gene duplications appears to be somewhat more frequent in eukaryotes [17]?

Adaptive evolution in prokaryotes is promoted by at least three main factors: first, a high strength of selection relative to eukaryotes, on account of their generally large population sizes [18]; second, their ability to obtain genes by horizontal gene transfer (HGT), which enables the sharing of niche-relevant functions between distantly-related microbes living in the same environment [19]; and third, their use of stress-induced hypermutation [20], which may increase the production of adaptive variants as a “last gasp” response to a challenging environment.

Although we know that these processes can drive ecological adaptation in prokaryotes, identifying the fraction of genetic variation that is associated with these functional changes remains a challenging problem. In the case of bacteria, whole-genome analyses must take into account widespread HGT, which means that different genes often disagree on the overall species tree [21]. This is a considerable problem for analyses of functional divergence, which require a tree in order to determine the branch upon which a particular trait arose.

The rationale for previous methods to identify functional divergence, and indeed the new approach described here, derives from the neutral theory of Kimura [22] (1983), which predicts that residues important for the function of a protein will be under strong functional constraint and therefore evolve slowly. These considerations have motivated the development of a number of methods for identifying changes in selective constraints on protein-coding genes and on single amino acid sites and lineages in a phylogenetic tree [23], [24], [25], [26], [27], [28], [29], [30], [31], [32], [33]. At the protein level, Gu [34], [35], [36] developed a Bayesian approach to identify functional divergence, which has become the most widely used. Comparisons of amino acid site-specific evolutionary rate or residue conservation between two homologous clades can therefore be used to identify amino acid sites at which selective constraints have changed, potentially indicating functional divergence.

Recently, we have developed a new distance-based method which explores a bifurcating phylogenetic tree, testing for functional divergence at each node by comparing the two downstream clades to an outgroup in order to identify sites at which substitution rates per amino acid sites have shifted [11], [37]. Similar to other methods, our approach was limited to tests of one gene at a time, unless the phylogeny of all genes could be fixed in advance.

Because general patterns of functional divergence and their link to ecological changes cannot be understood by the analysis of single genes, in the present study, we have optimised our method to (i) handle analyses of functional divergence that include hundreds of complete proteomes, (ii) address the fact that the phylogenies of individual proteins do not necessarily agree with the true phylogeny, as is often the case with organisms that acquire genes through HGT, (iii) provide an intuitive probability assignment for each test which takes the underlying phylogeny of the sequences into account and (iv) explore all levels of each gene tree, testing for functional divergence at each node.

Using this novel method to detect functional divergence, we infer patterns of radical change for each protein individually, and then cluster species according to the functional categories (derived from COG [38]) in which they exhibit significant evidence of functional divergence. We provide a fast, open source implementation of our method in the C++ program *CAFS* (Clustering analysis of functional shifts). We perform an analysis of functional divergence on 750 bacterial proteomes. This set includes bacteria from various different ecological niches and therefore provides a good dataset for identifying ecology-related functional divergence. Our approach (i) reveals striking patterns of convergent evolution in phylogenetically distinct but ecologically related groups of bacteria, including pathogens, endosymbionts, and thermophiles, (ii) provides additional support for the view that bacteria have a conserved set of core functions, with a more variable metabolic layer and (iii) provides a detailed picture of how individual species of unusual bacteria have diverged from their closest relatives.

## Results and Discussion

### A conserved functional core and variable crust in the evolution of bacterial proteomes

An obvious sign of functional divergence (also understood here as changes in substitution rates per amino acid site in proteins) would be a set of homologs that spans multiple COG categories. In this study we focus only on those alignments where all sequences have the same COG annotation. This represents the majority of homologs and is a reflection of the relatively broad character of the COG categories.

**Figure 1:**
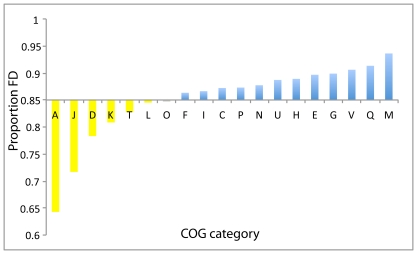
Different categories of genes experience different levels of functional divergence. Proportion (FD) is the proportion of tested branches with at least one functionally divergent site across all gene trees in a particular functional category. Categories are labeled according to the COG ontology system [38], [73]. Eighteen of the nineteen categories fall into two groups: significantly enriched or impoverished. Most information processing genes (K, J, L, A) fall into the latter group, while metabolic functions (E,F,G,H,P,Q) and genes involved in defense (V) or found on the cell surface (M) are enriched for radical change. Description of COG tags can be found in Table S5.

The kinds of functional shifts that we detect on the basis of conserved, radical amino acid substitutions are therefore subtler and not noticeable from simply comparing the COG classifications across homologous sequences. We used chi-squared tests to evaluate the differences in functional divergence between COG gene categories in our dataset (see Figure 1). We compared the proportion of positive tests for functional divergence within each of the 19 COG categories to the background expectation, which was calculated by combining all categories. If genes in different functional categories have similar propensities to undergo functional divergence, we would expect the proportion of positive tests in each category to be similar to the mean, resulting in few significant cases of enrichment. However, eighteen of the nineteen categories were either enriched or impoverished for functional divergence, while only one category failed to deviate significantly from the background expectation.

To test whether this polarization of our dataset was simply due to an artifact – for instance, the use of a non-conservative enrichment test – we performed simulations in which the genes in our original dataset were randomly assigned to one of the 19 COG categories before testing for enrichment. In these simulations, events of functional divergence were much more evenly distributed among the categories, so that 93% of categories were neither enriched nor impoverished for functional divergence relative to the background level. This result indicates that the probability of functional change is not evenly distributed among the real categories: there is a stark division between enriched and impoverished categories. This supports the idea that bacterial proteomes comprise a relatively unchanging core (that is, genes in impoverished categories) coupled with a set of more variable functions (enriched categories), as previously noticed [39], [40], [41].

The impoverished categories are almost exclusively those involved with information storage and processing, including DNA replication, recombination, and repair (L); transcription (K), ribosome biogenesis (J); and cell division (D). Metabolic genes were among those enriched for functional divergence, including genes involved in the metabolism of coenzymes (H), secondary metabolites (Q), carbohydrates (G), amino acids (E) and nucleotides (F). Along with these metabolic categories, cell wall and envelope genes (M) and cellular defense mechanisms (V) were among the most enriched categories in our analysis, highlighting the critical role of the environment in directing lineage-specific episodes of functional change. Taken together, our results agree with a number of previous reports indicating that proteins involved in information processing are more conserved across large evolutionary distances than those involved in metabolism [39], [40], [41], [42].

An additional point bears emphasizing here: since our method controls for the level of conservation at each node on the tree, the significance of a particular substitution pattern depends on the background evolutionary rate so that in slow-evolving proteins, relatively conservative substitutions are detected as significant events of functional divergence, whereas only very unusual substitution patterns will attain significance in fast-evolving proteins. Therefore, our results indicate that information processing genes are not only more conserved than others purely in terms of evolutionary rate, but that they also experience less functional change even taking this low rate of sequence evolution into account.

Why are informational genes under greater functional constraint than the rest of the proteome? One possibility, which follows Crick's concept of the “frozen accident” [43], is that too many other genes depend on the basic functions of translation, transcription, and repair: functional changes in these genes would disrupt many other systems in the cell. This hypothesis is supported by the observation that the COG category containing protein trafficking and chaperones (O) is also impoverished: the core activities of generalist chaperones such as GroEL and DnaK are required for the proper folding of many different proteins in bacterial cells [44].

### Host interactions constrain functional change in pathogenic and symbiotic bacteria

Does the ecological niche of an organism influence the pattern of functional change it experiences? To answer this question, we evaluated the enrichment of functional divergence in each species relative to the others in our dataset. To calculate the enrichment status of each species, we used the same statistical strategy as employed for enrichment by functional category: we calculated a background proportion of successful tests for functional divergence over all species, and then compared this to the proportion for each species individually using chi-squared tests (a full table of these results can be found in Table S1). We also used chi-squared tests to identify associations between these three enrichment patterns (enrichment, impoverishment, or neither) and organism lifestyle, as is summarized in Table 1. While there was no statistically significant difference between psychrophiles and mesophiles in terms of functional divergence (chi-squared = 0.9762, P = 0.6138), nor indeed was there significance when comparing thermophiles to free living bacteria.

**Table 1:** Effect of organism lifestyle on functional divergence.

Lifestyle	Comparison	Enriched	Neither	Impoverished	Significance
Psychrophile	Mesophile	2/61	6/433	1/66	*N.S.*
Thermophile	Mesophile	7/61	22/433	1/66	*N.S.*
Pathogen	Non-pathogen	22/77	272/294	47/38	*** (−)
Intracellular pathogen	Other pathogen	0/22	26/246	4/43	*N.S.*
Symbiont	Non-symbiont	4/95	36/530	13/72	*
*Intracellular endosymbiont	All others	4/224	14/360	15/133	*** (−)
All interactors	Free-living	44/55	410/156	70/15	*** (−)

Associations between lifestyle and enrichment for functional divergence: the numbers of genomes in each category are given in the form Lifestyle/Comparison. Significance was assessed with Yates-corrected chi-squared tests, or Fisher tests when the expected count was lower than 5 for any one cell in the contingency table. Significance codes: *N.S.* = P>0.05;

* = P<0.05,

** = P<0.01,

*** = P<0.001.

If an association was significant, “+” or “−” denote the direction of the shift associated with the lifestyle being tested. For instance, interactors are significantly impoverished (−) compared to free-living bacteria.

Thermophiles did however present a contrasting pattern of functional divergence in comparison to the general pattern, with two COG categories being enriched for functional divergence in thermophiles while being impoverished in general. These categories are directly related with the survival of cells under heat stress: category K, which comprises mostly transcription factors, and category L, which is involved in DNA replication, recombination and repair. Above certain temperature threshold, molecular pathways undergo dramatic temperature induced alterations that drive to cytotoxicity, radiosensitization and thermotolerance [45], [46]. Among all the responses that take place in the cell under high temperatures, inhibition of DNA, RNA and protein synthesis is the response that involves a complex and fine-tuning of regulation mechanisms, mainly orchestrated by transcription factors [46]. One such important regulated mechanism is the induction of heat-shock proteins, particularly involved in mitigating the cytotoxic effects due to the non-specific aggregation of unfolded and denatured proteins [47], [48].

Interestingly, we found that all bacteria that interact with a host as an integral part of their lifestyle (including pathogens, parasites, symbionts and commensals) were significantly impoverished for functional divergence in comparison to their free-living relatives (see Table 1). This result is somewhat surprising because pathogens and symbionts generally experience higher rates of evolution than free-living bacteria, although much of the increase can be attributed to heightened genetic drift [9]. Our results suggest that once the overall conservation level of proteins is accounted for, these bacteria have undergone less functional change than their free-living relatives. This result can be explained by greater ecological constraints on host-associated bacteria, which must adapt to the highly specific environment of their host. In particular, pathogenic and symbiotic bacteria preferentially lose metabolic genes as they no longer require the capacity to exploit as wide a range of nutrient sources as free-living bacteria [9].

Since these are precisely the kind of genes that are most amenable to functional change (Figure 1), their loss from host-associated bacteria explains the relative impoverishment of functional divergence in these proteomes. The remaining genes are also under strong constraints imposed by the specialized environment they are in, limiting therefore any opportunity for functional divergence (Toft and Fares 2008, 2009). However, variability in genome size is a complicating factor in this analysis because host-associated bacteria tend to have smaller genomes than their free-living relatives. For instance, endosymbiotic bacteria of insects underwent substantial reduction in the gene content, with genomes sizes ranging between 144 kb and 792 kb depending on the host (in comparison, *E. coli* K12 has a genome size of 4.639 Mb) (See for example [49], [50], [51], [52], [53], [54], [55]. Since functional divergence often follows gene duplication [1], it might be expected that larger genomes would be enriched for new functions in comparison to smaller ones.

Does genome size alone account for the observed differences between host-associated and free-living bacteria? To test this possibility, we modeled genome enrichment and impoverishment for functional divergence as a function of lifestyle (host-associated vs. free-living) and genome size (in nucleotides) using a generalized linear model, a saturated model was fit using the glm function in R (R Development Core Team, 2010), with enrichment or impoverishment for functional divergence as the response variable (binomial errors), and genome size (continuous, bp) and lifestyle (categorical, free-living or host-associated) as the explanatory variables. This was simplified to a minimal adequate model using the step function. The interaction between genome size and lifestyle was non-significant and was removed during model simplification (see Table S2). Both lifestyle and genome size were significant, with host-associated bacteria significantly more likely to be impoverished (P = 1.28×10^−13^) and, perhaps surprisingly, a modest tendency towards impoverishment in larger genomes (P = 0.03). Therefore, variation in genome size does not account for the observed differences in functional divergence between host-associated and free-living bacteria.

To better define the effect of lifestyle on functional divergence, we identified the functional categories with the greatest consistent differences in enrichment status between host-associated and free-living bacteria. Interestingly, genes involved in vesicular transport and secretion systems (U) were enriched for functional divergence in host-associated bacteria but neither enriched nor impoverished in free-living bacteria, while signal transduction genes (T) were impoverished in host-associated bacteria but enriched in their free-living relatives (Table S3). This pattern can be readily understood in terms of the lifestyles of host-associated bacteria, as pathogens use elaborate secretion systems for delivering toxins and other virulence factors to their host [56], while symbionts provision their hosts with nutrients as part of their mutually beneficial relationship [57], [58]. In addition, the impoverishment in host-associated signal transduction genes reflects their adaptation to a relatively constant host environment, which is considerably more stable than the fluctuating conditions experienced by their free-living relatives.

What is the relative importance of genome size variation compared to functional divergence in bacterial adaptation? This question is difficult to answer because both of these evolutionary phenomena are at work in the process of ecological bacterial adaptations. Therefore, micro-evolutionary processes, such as functional divergence, necessarily accompany macro-evolutionary processes, such as genome shrinkage or HGT, during bacteria adaptation to different ecological conditions. The timing and relative importance for each of these phenomena is, nevertheless, varied over the different stages of adapting to a new environment. Taking the example of symbiotic bacteria of insects, these bacteria are characterized by a dramatic genome streamlining, high mutation rates and the functional divergence of genes involved in an endosymbiotic lifestyle. When did these processes occur? We predict that HGT was important for the free-living ancestor of these bacteria to acquire pathogenic genes and invade the eukaryotic cells of the host. Dramatic gene loss in these invading bacteria may have become the next important evolutionary leap, making bacteria dependent upon the host. Finally, functional divergence may have contributed importantly to the refinement of the adaptation of these bacteria to the novel ecological conditions.

### From proteome-wide to residue-level functional divergence

**Figure 2:**
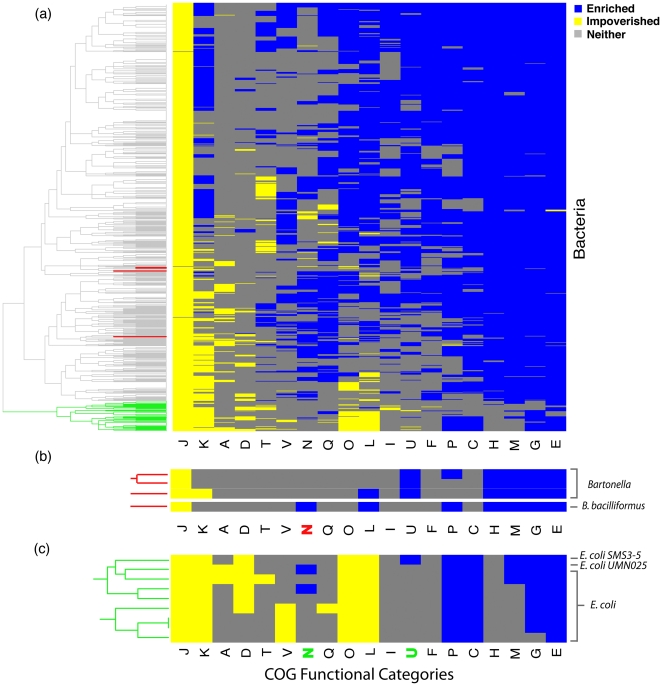
Visualizing high-level patterns of functional divergence. We used hierarchical clustering to reveal the main patterns of functional divergence in our dataset of 750 bacterial proteomes. (a) The complete heatmap, with a dendrogram corresponding to category clustering, and species clustering along the left hand side. Visualizing the data in this way reveals the extreme impoverishment of proteins involved in ribosome biogenesis (J), as well as the enrichment of categories involved in interaction with the environment (E, M, G, H, C, P) across all species. (b) Lineage-specific events of functional divergence picked out from the heatmap (dendrogram colors denote the regions expanded upon – a larger version of the complete heatmap is available as Figure S1). Unlike other *Bartonella* species, *B. bacilliformus* is impoverished for divergence in cell motility genes (N), and is unique among *Bartonella* species in using a flagellum to infect erythrocytes. (c) Two strains of *E. coli* – SMS 3–5 and UMN026 – have phylogenetically atypical patterns of functional divergence: the constraints on cell motility (N) are among those that have relaxed relative to the other strains in SMS 3–5, while UMN026 is uniquely enriched for secretion system (U) genes.

In order to visualize the results of our functional divergence analysis, we performed two-dimensional hierarchical clustering on the enrichment status (enriched, impoverished, or neither) associated with each species and functional category – that is, we clustered species according to similarities in their enrichment status across the 19 functional categories, resulting in the heatmap and dendrogram in Figure 2a (complete dendogram and heatmap is available in Figure S1). This is a powerful and intuitive way to represent our results because it reveals the overall patterns in the data – such as the extreme conservation among informational genes, particularly those involved in ribosome biogenesis (J) - while also highlighting individual, lineage-specific exceptions to the general trends. In this section, we demonstrate the utility of this approach by using the heatmap to identify species that have undergone major functional shifts.

Although top-level bacterial groups (such as the divisions of the proteobacteria, the Firmicutes, Actinobacteria, and so on) are not resolved in our dendrogram of functional divergence (Figure 2), family and genus-level relationships often are, presumably because of close phylogenetic relatedness, shared gene content, and similarity of ecological niche. This allows us to identify individual species with atypical patterns of functional divergence. A particularly striking case is that of the *Bartonella* genus (Figure 2b), which are a group of intracellular parasites that infect and replicate in erythrocytes [59]. Of the four *Bartonella* species in our dataset, only one – *Bartonella bacilliformus* – is enriched for functional divergence in cell motility genes (N), with the others being impoverished (2 species) or neither enriched nor impoverished (1 species).

Remarkably, this is the only member of the genus that possesses flagella [60]. Since erythrocytes lack an active cytoskeleton, they cannot be induced to take up external bacteria by invagination [61]. Instead, erythrocyte invasion by *Bartonella* species is an active process [62]. The mechanism employed by *Bartonella bacilliformus* involves the use of its flagella [63] and is more efficient than that of other *Bartonella* species, with up to 80% of erythrocytes infected [62], [64]. This appears to be a clear case where our approach has identified an interesting, lineage-specific case of adaptation to a specialized ecological niche.

Our heatmap turns up surprises even among relatively well-characterized species (Figure 2c). As expected, closely related *E. coli* and *Shigella* strains cluster together at the bottom of the dendrogram (Figure 2a). *E. coli SMS 3–5*, a multidrug-resistant, heavy-metal tolerant strain isolated from a polluted industrial environment [65] is distinguished from other *E. coli* strains on the basis of a relaxed functional constraint in the category of cell motility (N); most others are impoverished for functional divergence, while *SMS 3–5* is enriched. This profile correlates well with what is known about the biology of this strain, which is unique among sequenced *E. coli* genomes in possessing a second, intact lateral flagellar system called Flag-2, in addition to the normal peritrichious flagella found in other *E. coli* strains [65], [66]. This system was originally characterized in a different strain, *042*, where it has been rendered nonfunctional by a frameshift mutation in one of the component genes [66], although it appears to be complete in *SMS 3–5* [65].

**Figure 3:**
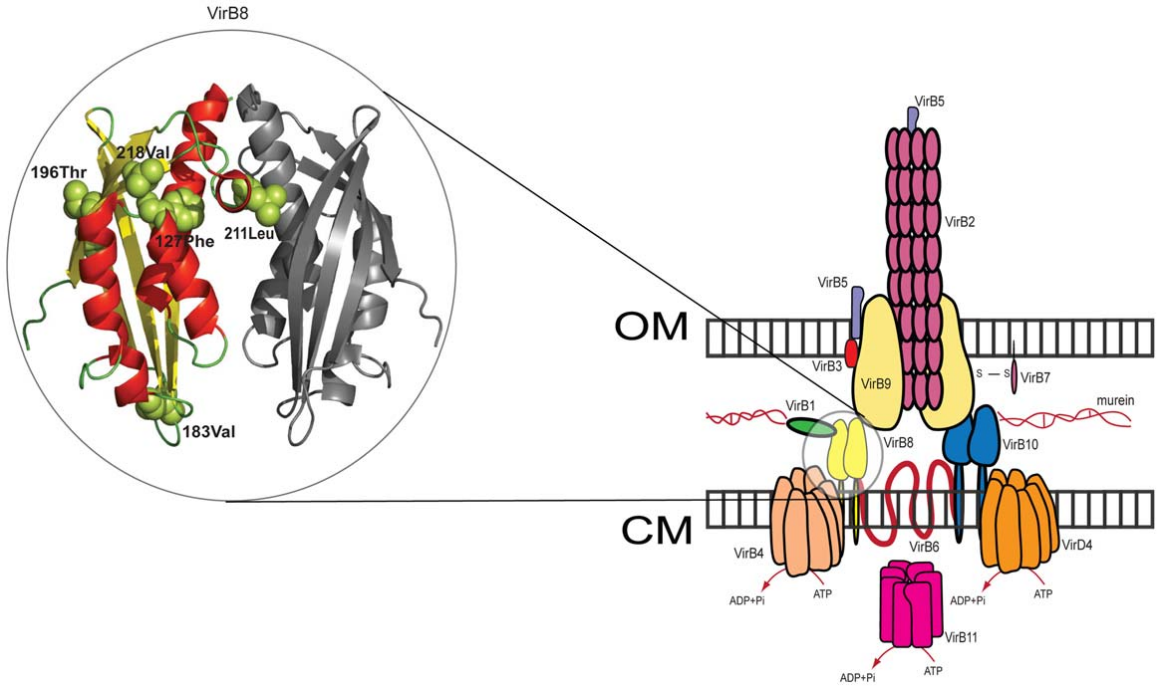
Amino acid residues under functional divergence in *E. coli* UMN026 VirB8. *Right*: the structure of a Type IV secretion system found only in two strains of *E. coli*. CM = cytoplasmic membrane, OM = outer membrane. The complex structure is based on that of Baron (2006) [84]. In UMN026, the central complex proteins VirB8 and VirB9 are under functional divergence. *Left*: Of the five sites detected by CAFS that could be mapped to the VirB8 crystal structure [69], there is evidence that four are involved in forming the VirB8 homodimer, suggesting that functional divergence at these positions is involved in altering the quaternary structure of the complex.

Another *E. coli* proteome with an unusual pattern of functional divergence is O17:K52:H18 (strain UMN026), a multidrug-resistant strain that causes urinary tract infections [67]. Unique among *E. coli* and *Shigella* species, this strain is enriched for functional divergence among genes involved in secretion (U). Investigation of the genes underlying this enrichment revealed functional divergence in the VirB8 and VirB9 genes, which encode core proteins in a Type IV secretion system found only in two *E. coli* strains – UMN026 and 018 (ED1a), although the latter species is not enriched in this category. In other bacteria, Type IV systems are involved in the exchange of DNA with the environment, as well as the delivery of effector proteins to host cells [68]. Since these two proteins are important components of the Type IV secretion systems of other bacteria, functional divergence in these genes may be involved in adapting the system to an UMN026-specific role (see Figure 3).

To gain further insight into the possible implications of the UMN026-specific changes in these proteins, we mapped the specific residues under functional divergence in VirB8 (also output by *CAFS*) onto the *Agrobacterium tumefaciens* crystal structure [69]. Of the 14 sites under functional divergence (see Table S4), 5 could be mapped onto the crystallized region of the protein. Of these 5, 4 are at or close to positions previously shown to be of functional importance. Thr-196(residues numbered according to the *A. tumefaciens* sequence), which CAFS detected as being under functional divergence in UMN026, is directly involved in the stabilization of the VirB8 homodimer [69], as is Leu-211, another functionally divergent site. Additionally two sites identified by our approach are at positions that suggest they may have an indirect role in dimerization. Val-218 is located between two other residues (Leu-217 and Val-219) that are involved in dimer formation, while Phe-127 is adjacent to Ser-128, a conserved residue that stabilizes the interaction surface on VirB8. The function of the other site detected under functional divergence, Val-183, is currently unknown. Taken together, these results indicate that functional divergence in *E. coli* UMN026 VirB8 has occurred at residues important in forming the homodimer, which may have important implications for the overall structure and function of the complex. With no crystal structure available for VirB9, it is more difficult to evaluate the functional significance of the sites detected there. Further, we detected functional divergence on 19 branches of the VirB9 tree, suggesting that this protein experiences a more general pattern of radical change.

## Methods

### Design and implementation

**Figure 4:**
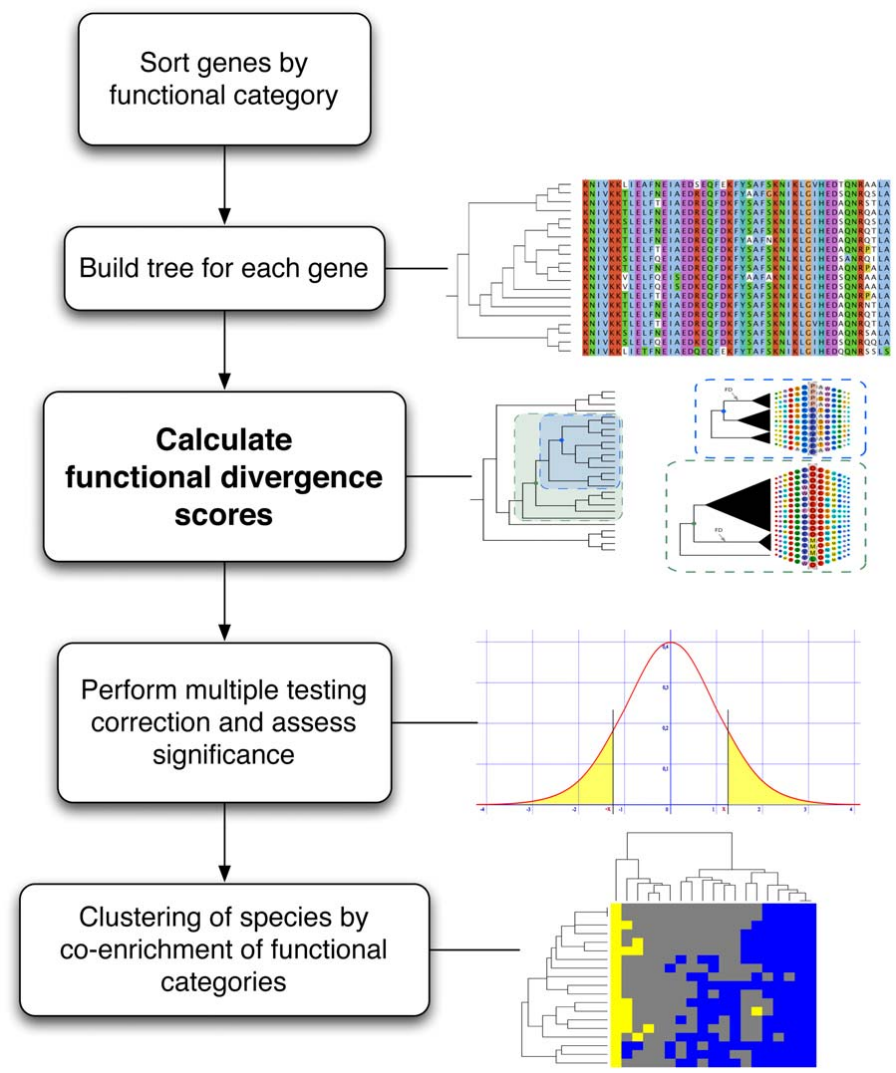
*CAFS* program workflow. After alignments have been built for each gene in the analysis, the alignments are sorted by functional category. In this case, the COG system was used [38], but any other ontology can be used as well. Trees are built for each gene using BIONJ [76] and the JTT substitution model [77], and sites are scored for functional divergence on each branch. Significance is assessed by simulating a distribution of test scores under a model of neutral evolution, taking the real phylogeny into account and using the False Discovery Rate approach to correct for multiple testing. For each species and functional category, we use chi-squared tests to evaluate whether the species is enriched or impoverished for functional divergence in that category, and then cluster species according to similarities in their profile across all 19 categories. This approach enables us to account for HGT while identifying interesting and atypical patterns of functional change in the data, as discussed in the main text.

Our analysis of functional divergence, the individual steps of which are detailed below, is summarized in Figure 4.

### Sequences, orthology, and alignment

The first step in a whole-proteome analysis of functional divergence is the grouping of orthologs within the species of interest. We leave orthology assignment for which a number of tools are already in use [70], to the users' choice according to their own needs. For the present analysis, we retrieved pairwise orthology assignments for 750 completely-sequenced bacterial genomes from the OMA database [71], [72], representing all bacterial data in the October 2009 revision of the database.

We chose the OMA project for its very broad phylogenetic coverage, as well as the favourable performance of its algorithm against other current orthology assignment methods [70]. In addition to providing pairwise orthology calls, the OMA algorithm assembles strict orthologous groups in which every member is directly orthologous to every other. The rationale for this strict approach to grouping is the exclusion of paralogs, which is important for a number of potential applications of the OMA database, such as phylogenetic analysis. Unfortunately, these groups are unsuitable for functional divergence analysis across large phylogenetic distances because lineage-specific gene duplications tend to break up genuine orthologs into multiple, overlapping groups (that is, clustering problems arise because pairwise orthologies are not necessarily transitive). Using these groups in our analysis would result in multiple testing of the same clade, each time with overlapping but incomplete sampling of downstream sequences. The inclusion of both orthologs and lineage-specific paralogs in the same group is, however, of no concern in our per-species comparison of divergence between different functional categories of genes, because our method relies on individual gene trees and not a single “species tree” to detect functional divergence (see below).

Therefore, we decided to build our own groups from the pairwise homology assignments in OMA, with the less stringent requirement that any sequence in a group be connected to at least one other sequence by pairwise homology. This strategy produces groups containing all orthologs and paralogs for a given gene, as appropriate for analysis of functional divergence. However, the approach is vulnerable to erroneous homology calls in the original database, because a single false call will cause two unrelated groups of sequences to be merged.

**Figure 5:**
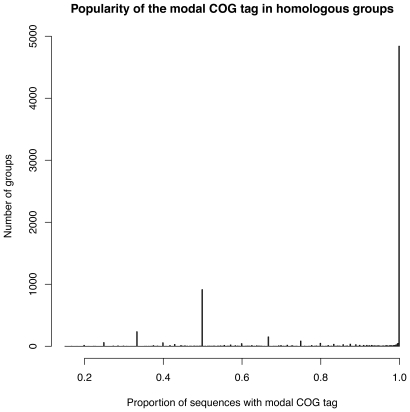
Number of COG assignments (tags) for each group of homologous sequences. We included both orthologs and paralogs in our sequence groups, because we are interested in functional divergence. The majority of groups consisted of sequences that had all been assigned to the same COG category, suggesting our grouping strategy did not lump together unrelated sequences due to spurious orthology calls. Since COG categories are relatively broad, we do not generally expect functional divergence to cause a sequence to shift from one category to another, an hypothesis that is also borne out by the clustering of related sequences within the same category. In our study, to avoid ambiguity we only use alignments in the group with a single COG tag.

To assess the possible effect of false OMA homology assignments on our dataset, we used the relevant genomic data at NCBI to assign COG ontology tags to each sequence [38], [73]. We then calculated the frequency of the modal COG tag in each group (see Figure 5). The largest group (4,788 alignments) contained only one COG tag each, validating our approach to grouping homologs (since COG categories are relatively broad, related sequences are expected to be annotated with the same tag). To avoid ambiguity in the clustering of functional categories, we only analyzed these single-tag alignments. We then filtered out poorly-characterized groups (annotated with the ambiguous R or S COG categories) and any group containing less than 9 sequences (one outgroup and 4 sequences downstream of the inner nodes), which we chose as the minimum number required for analysis (both as a requirement for stringency and also comparison to similar software Gu 1999). The final dataset comprised 3,813 groups, which were then analyzed with our novel approach (CAFS: Clustering Analysis of Functional Shifts). Other functional classifications such as Gene Ontology (GO) can also be used. However, caution is required because GO contains overlapping categories and alignments with multiple tags can lead to ambiguous results.

Sequence alignments were built for each group with MUSCLE [74], using the default parameters. Data on the ecological niches occupied by the species included in the analysis was retrieved from HAMAP [75] and from the Genome database at NCBI.

A typical alignment of 78 sequences takes 2 minutes and 40 seconds to analyze for functional divergence using CAFS on a standard desktop computer, including NJ tree-building. At the other extreme, the large-scale analysis reported below (44,416 tests of functional divergence/3,813 alignments) took 92 hours on a 40-node cluster.

### Building gene trees

When analyzing entire proteomes for functional divergence, the use of a species tree to infer events on each branch is problematic: extensive horizontal gene transfer (HGT), particularly among prokaryotes, means that genomes may not be related in a tree-like way [21]. We therefore calculated a tree for each gene (set of homologous sequences) in the dataset using BIONJ [76] (see Text S1 and Table S6 for a justification of the use of BIONJ and comparison with the use of maximum-likelihood trees), under the JTT model of protein sequence evolution [77] along with a gamma distribution(n = 4, alpha = 1.0) to correct for among sites variation of evolutionary rates(a fixed alpha value was used because of the time constraints involved in assessing alpha and other parameters for all alignments). Calculations for that gene were then made exclusively using the resulting tree.

### Scoring functional divergence

We here define functional divergence as the potential departure of the derived protein function from its ancestral one as a result of amino acid changes at important functional sites. Therefore functional divergence is detected on the basis of shifts in substitutution rates per amino acid site in proteins. This analysis of functional divergence can be used to provide a list of candidate genes for further experimental testing. Our method identifies amino acid sites within a protein, which have radical substitutions between clades and are statistically significant. This is carried out in each of the lineages of a tree, with each lineage being a cluster of 4 or more sequences.

The method steps through the phylogenetic tree and calculates functional divergence scores at each of the inner nodes. For each site of the protein, our approach compares the amino acid composition between two clades to that of an outgroup. This comparison is performed using BLOSUM62 amino acid substitution matrix [78], indeed any substitution matrix can be used. BLOSUM62 and related matrices provide an empirical measure of the likelihood of the transition of one amino acid to any of the other 20 (including its conservation). Scores of functional divergence (*FD_score_*) for each column are given by:
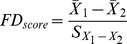
(1)where 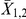 are the mean substitution scores for the transition from clades on either side of the bifurcation in the phylogenetic tree relative to the outgroup and 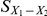, the standard error for unequal sample sizes with unequal variances, is given by:
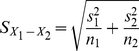
(2)


### Significance testing

To test the significance of functional divergence events, we simulated multiple sequence alignments of the same size as the real alignment but in which proteins were evolved under a neutral evolution model. Because functional divergence was tested in protein alignments, the seed ancestral sequence was protein based and this evolved under the JTT model.

For our simulations, we used the gene-specific tree topology and branch lengths calculated above. We built at least 1000 such simulated alignments (more simulations are created if mean and standard deviation have not converged within a difference of 1*10^−6^ after 1000), in each of which we searched for functional divergence and calculated a score according to equation (1). To allow for the gaps in the sequences we simulated alignments with the number of columns equal to the length of the input alignment minus the average number of gaps in each species. This search resulted in a null distribution of the test score against which P-values for the real data were calculated. These values were then corrected for multiple testing by the False Discovery Rate method [79] using an alpha value of 5% as the threshold of significance. Following this procedure, branches on the tree that still possess at least one significant amino acid site were considered to be under functional divergence for the purposes of enrichment and clustering.

### Enrichment analysis

Once all alignments were analyzed, we performed three different enrichment tests to ask three different biological questions. These are based on a chi-squared test:
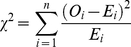
(3)Where O_i_ is the observed frequency of genes/alignments under functional divergence, E_i_ is the expected frequency and n is the number of possible outcomes of each event. We used the enrichment tests to identify (i) species and (ii) categories of genes that experienced significantly more (enriched: O_i_ – E_i_>0) or significantly less (impoverished: O_i_ – E_i_<0) functional divergence when compared to the background level (that is, P<0.05 in a chi-squared test). We then calculated (iii) the enrichment status of each category within each species, in order to identify lineage-specific shifts in the pattern of functional divergence. It should be noted that the chi-squared test used scales for the size of each of the groupings considered in our results.

### Hierarchical clustering

We created a heatmap from the enrichment status of functional categories within species to help visualize the structure in our large dataset. To do this we used the *heatmap.2* function from the *gplots* library in R (R Development Core Team, 2010). This function performs two-dimensional hierarchical clustering according to Euclidean distance and outputs a heatmap together with a corresponding dendrogram. Visualizing the results of the analysis in this way allows identifying unusual patterns of functional divergence in particular functional categories or convergent functional divergence among phylogenetically unrelated sequences.

### Implementation

CAFS was implemented in C++ and is available under the GNU General Public License v.3 for Linux, Mac and Windows. The code was written using the GNU Scientific Library and the Bio++ libraries [80]. The program is accompanied by full documentation and enables the user to perform several different kinds of analyses, including the identification of lineage-specific functional divergence in a gene-of-interest (such as that reported by Williams et al. (2010) [37]) and the kind of multi-proteome investigation reported here. The latest version of the code and documentation is available at http://bioinf.gen.tcd.ie/~faresm/software/software.html.

### Comparison to other applications and assessment of Error

As mentioned previously many programs have been developed to predict putative sites of functional divergence in a multiple sequence alignment. Of those, the most commonly used method is DIVERGE [81], therefore we used this as a benchmark. We found that our method could analyze large alignments, automate proteome scale analyses and performs analyses on tagging systems which DIVERGE does not (Also see Table S7, Text S2, Table S8 and Text S3 for a detailed comparison of our method with Diverge). Our method also performs simulations, which scales the cutoff value for each alignment, this scaling allows us to analyze all homologs and hence is not limited to orthologs. Our program automates the building of trees with a more detailed model of evolution and better tree building algorithm. Given that the tree building algorithm is distance based we performed a comparison to trees built using RaxML [82], 92% of the sites identified using the distance based trees were identical to those using the maximum likelihood method. Given the differences between our software and that of DIVERGE it is very difficult to make comparisons in terms of the sites reported by each program. DIVERGE compares two clades after a duplication event and our software compares two clades and an outgroup. We do so in order to assess events of functional divergence at any testable node on a tree and also because we feel it provides stronger evidence of functional divergence. Another widely used software to identify the strength of selection in protein-coding genes is PAML. This package employs the programs yn00 and codeml for testing the D_n_/D_s_ ratio. This means PAML works on nucleotide level while CAFS uses protein sequences, which gives it the ability to assess divergences of greater magnitude. An additional feature of our program is the ability to easily automate and carry out large analyses, as shown in this study. Another philosophical difference between both approaches is that, to detect positive selection, using programs like PAML a strong signal is required, which would make it difficult to detect episodic positive selection. CAFS only requires an amino acid substitution fixed by positive selection in a functionally important region of the protein followed by strong purifying selection.

Given the difficulty of finding definitive positive controls for an analysis of this nature we felt it important to demonstrate that there would not be a large false positive rate. We simulated 20 alignments under a codon model with neutral evolution (non-synonymous, Ka, to synonymous, Ks, rates ratio ω = Ka/Ks = 1) using the evolver package in PAML [83]. These alignments were converted to amino acids and analysed with CAFS under the default alpha value of 0.05, with this value the expectation would be 5% of sites being reported as functionally divergent. Our software reported an average of 2.3% of sites as functionally divergent. Given this percentage we are confident that our methodology of significance testing and implementation of false discover rate is not susceptible to a large number of false positives. Further details of this section can be found in Text S1 and S2 and Tables S6 and S7.

In conclusion, The identification of functional divergence and ecological adaptation from sequence data is an interesting and important goal in evolutionary biology, with the potential to deepen our understanding of the evolution of individual traits and species, as well as the processes of evolution as a whole. Bacteria display an astonishing capacity for adaptation to different lifestyles and ecological niches, but investigating the evolution of these traits is problematic because their phylogenetic context is often unclear.

Here, we have circumvented this problem by evaluating functional divergence on gene trees and then clustering species by gene functional category. Our approach revealed the overall patterns that have characterized functional change during the evolution of bacteria, including strong constraint on information storage and processing genes and also constraints induced by the host on pathogenic and symbiotic bacteria. It also identified lineage-specific events of atypical functional divergence, such as the use of flagella by *Bartonella bacilliformus* to invade host erythrocytes and residue-level changes in the VirB8 protein of *E. coli* UMN026. This is, to our knowledge, the first method that can be used to identify functional divergence at the level of entire proteomes. Although used here to perform a large-scale analysis on bacteria with the use of COG categories, our CAFS software is extremely flexible and can be applied to individual genes, lineages, or groups of proteomes using any ontology system in order to investigate functional divergence at every level of biological organization.

## Supporting Information

Figure S1 **Visualizing high-level patterns of functional divergence.** We used hierarchical clustering to reveal the main patterns of functional divergence in our dataset of 750 bacterial proteomes. The complete heatmap, with a dendrogram corresponding to category clustering, and species clustering along the left hand side. Visualizing the data in this way reveals the extreme impoverishment of proteins involved in ribosome biogenesis (J), as well as the enrichment of categories involved in interaction with the environment (E, M, G, H, C, P) across all species.
(TIFF)

Table S1 **Enrichment of COG gene categories for functional divergence.** The annotation for each COG category was retrieved from http://www.ncbi.nlm.nih.gov/COG/. Enrichment was evaluated with Chi-squared tests. “Not enriched” indicates there was no significant association between the number of lineages under functional divergence and the COG category. Impoverishment and Enrichment denotes the direction of a significant association.
(DOCX)

Table S2 **Enrichment status of 750 bacterial species.** Enrichment status was calculated with a Chi-squared test based on the number of testable branches on our gene trees where a particular species was under functional divergence.
(DOCX)

Table S3 **Effect of bacterial lifestyle and genome size on functional divergence.** We used a generalized linear model with binomial errors to assess the impact of lifestyle and genome size on the enrichment and impoverishment of genomes for functional divergence. The saturated model was fit with the *glm* function in R, and simplified to a minimal adequate model with the *step* function, which determined that the interaction was not significant. Both lifestyle and genome size have a significant impact on enrichment status, with host-associated bacteria and bacteria with larger genomes more likely to be impoverished for functional divergence.
(DOCX)

Table S4 **Enrichment status of gene categories in host-associated and free-living bacteria.** Categories U and T show different levels of enrichment for functional divergence when the analysis is run on these groups of bacteria independently.
(DOCX)

Table S5 **Sites under functional divergence in VirB8.** The left column shows the sites found in E.coli UMN026, the sites on the right show the homologous sites in *Agrobacterium tumefaciens*. Sites without a value for *Agrobacterium tumefaciens* represent sites, which have not been crystalised.
(DOCX)

Table S6 **Detecting functional divergence using BioNJ trees.** The number of FD sites predicted at the 0.05 p-value level using BioNJ trees is consistently smaller. This indicates a stricter scoring scheme, which potentially reduces false positives. It can be seen from the last column, that the percentage of the FD sites detected through BioNJ trees that are also detected through ML trees increases with larger number of sequences in the alignment and approaches 100%. Only the smaller alignments show noticeable discrepancies but some of these can already be explained by the effect of different tree topologies. Overall, the above results confirm the suitability of BioNJ for tree construction, particularly for alignments with a large number of sequences.
(DOCX)

Table S7 **Comparison of runtime for two methods of functional divergence.** The most widely used program for detection of functional divergence is DIVERGE [81]. Even though it is well-suited for individual analyses, it can not be used for a large-scale study such as the one presented here. This is because the size of alignments dealt with exceeds the limits of the data that DIVERGE can handle. It is also not designed to be run automatically.
(DOCX)

Table S8 **Functionalities of CAFS in comparison with DIVERGE.**
(DOCX)

Text S1 **Justification for use of BioNJ and Comparison of results performed with Maximum Likelihood trees vs BIONJ trees.**
(DOCX)

Text S2 **A Note about testable alignments and computation time for functional divergence detection.** It should be noted that since DIVERGE could not run the 4 largest alignments in this subset of our dataset, we predict that at least half of the alignments in our full dataset can not be analysed by the DIVERGE software. Indeed we were unable to run analysis on any alignment over 86 sequences long, however DIVERGE would read alignments up to 100 sequences long. Another further problem about the calculations performed with DIVERGE2.0 is the impossibility to perform analyses collected in Gu2001 which pertains to the method in [81]. This analysis did not work for any of the alignments above. All alignments failed with the error “Please recheck input sequence data and tree information”, for which we could not find documentation. In addition to the times stated above we would like to add that DIVERGE works on an alignment by alignment basis and as such the user must manually chose files to be analysed and also the trees to be input and the nodes to be tested. This makes proteome level analyses prohibitive. The runtimes given in the table above do not include the time it takes for the user to choose nodes for functional divergence testing with DIVERGE.
(DOCX)

Text S3 **Conclusion of Comparisons of the methods to identify functional divergence.** We conclude that whilst we understand and acknowledge the value of building maximum likelihood trees we feel that one of the biggest assetts of our program is speed and automation. With this in mind, we wish to demonstrate to the user that whilst BioNJ trees are calculated within the program the results recieved are ∼92% comparable to those returned after the maximum likelihood analysis. Given the high level of similarity between the maximum likelihood built trees and those built with BioNJ we feel that the conclusions drawn in the manuscript would hold in either circumstance. We also feel that analyses on the scale of those detailed in the main text are not possible for many who have limited computational resources. In reference to the DIVERGE comparisons we feel justified in our calculations that DIVERGE is not ideal for large scale analyses and can be troublesome to the user for even relatively small alignments. In addition to the tests run above we would like to say that on large scale analyses all of the information about the sites tested are collected and automatically analysed according to any tagging system applied to the dataset.
(DOCX)
