# Supplementary material for: Proteome-Wide Analysis of Functional Divergence in Bacteria: Exploring a Host of Ecological Adaptations
Source: PLoS One. 2012 Apr 26;7(4):e35659. doi: 10.1371/journal.pone.0035659 (PMC3338524; doi:10.1371/journal.pone.0035659)
Supplement: Table S1 — Enrichment of COG gene categories for functional divergence. The annotation for each COG category was retrieved from http://www.ncbi.nlm.nih.gov/COG/. Enrichment was evaluated with Chi-squared tests. “Not enriched” indicates there was no significant association between the number of lineages under functional divergence and the COG category. Impoverishment and Enrichment denotes the direction of a significant association. (DOCX) [file pone.0035659.s002.docx]

| Cog Tag | Functional category | Enrichment |
| --- | --- | --- |
| L | Replication, recombination and repair | Impoverished |
| D | Cell cycle control, cell division, chromosome partitioning | Impoverished |
| A | RNA processing and modification | Impoverished |
| J | Translation, ribosomal structure and biogenesis | Impoverished |
| K | Transcription | Impoverished |
| I | Lipid transport and metabolism | Enriched |
| T | Signal transduction mechanisms | Impoverished |
| N | Cell motility | Enriched |
| O | Posttranslational modification, protein turnover, chaperones | Not Enriched |
| U | Intracellular trafficking, secretion, and vesicular transport | Enriched |
| H | Coenzyme transport and metabolism | Enriched |
| Q | Secondary metabolites biosynthesis, transport and catabolism | Enriched |
| G | Carbohydrate transport and metabolism | Enriched |
| E | Amino acid transport and metabolism | Enriched |
| F | Nucleotide transport and metabolism | Enriched |
| C | Energy production and conversion | Enriched |
| P | Inorganic ion transport and metabolism | Enriched |
| M | Cell wall/membrane/envelope biogenesis | Enriched |
| V | Defense mechanisms | Enriched |
