# Supplementary material for: Proteome-Wide Analysis of Functional Divergence in Bacteria: Exploring a Host of Ecological Adaptations
Source: PLoS One. 2012 Apr 26;7(4):e35659. doi: 10.1371/journal.pone.0035659 (PMC3338524; doi:10.1371/journal.pone.0035659)
Supplement: Table S2 — Enrichment status of 750 bacterial species. Enrichment status was calculated with a Chi-squared test based on the number of testable branches on our gene trees where a particular species was under functional divergence. (DOCX) [file pone.0035659.s003.docx]

| ALISL | Impoverished |
| --- | --- |
| BARBK | Impoverished |
| BARHE | Impoverished |
| BARQU | Impoverished |
| BART1 | Impoverished |
| BAUCH | Impoverished |
| BLOFL | Impoverished |
| BLOPB | Impoverished |
| BRUA1 | Impoverished |
| BRUA2 | Impoverished |
| BRUAB | Impoverished |
| BRUC2 | Impoverished |
| BRUME | Impoverished |
| BRUO2 | Impoverished |
| BRUSI | Impoverished |
| BRUSU | Impoverished |
| BUCA5 | Impoverished |
| BUCAI | Impoverished |
| BUCAP | Impoverished |
| BUCAT | Impoverished |
| BUCBP | Impoverished |
| BUCCC | Impoverished |
| BURA4 | Impoverished |
| BURCA | Impoverished |
| BURCC | Impoverished |
| BURCH | Impoverished |
| BURCJ | Impoverished |
| BURCM | Impoverished |
| BURM1 | Impoverished |
| BURM7 | Impoverished |
| BURM9 | Impoverished |
| BURMA | Impoverished |
| BURMS | Impoverished |
| BURP0 | Impoverished |
| BURP1 | Impoverished |
| BURPS | Impoverished |
| BURTA | Impoverished |
| BURVG | Impoverished |
| CUPTR | Impoverished |
| ECO45 | Impoverished |
| ECO55 | Impoverished |
| ECO57 | Impoverished |
| ECO5E | Impoverished |
| ECO81 | Impoverished |
| ECODH | Impoverished |
| ECOL6 | Impoverished |
| ECOLI | Impoverished |
| ECOSE | Impoverished |
| ECOUT | Impoverished |
| HERAR | Impoverished |
| JANMA | Impoverished |
| LACH4 | Impoverished |
| METC4 | Impoverished |
| METEP | Impoverished |
| NITHX | Impoverished |
| NITWN | Impoverished |
| OLICO | Impoverished |
| POLNS | Impoverished |
| POLSQ | Impoverished |
| PSE14 | Impoverished |
| PSEA8 | Impoverished |
| RALEH | Impoverished |
| RALEJ | Impoverished |
| RALME | Impoverished |
| RALPJ | Impoverished |
| RALSO | Impoverished |
| RHOPA | Impoverished |
| RHOPS | Impoverished |
| SHIB3 | Impoverished |
| SHIDS | Impoverished |
| SHIFL | Impoverished |
| SHISS | Impoverished |
| SODGM | Impoverished |
| STRT1 | Impoverished |
| WIGBR | Impoverished |
| YERE8 | Impoverished |
| YERP3 | Impoverished |
| YERPA | Impoverished |
| YERPB | Impoverished |
| YERPE | Impoverished |
| YERPG | Impoverished |
| YERPN | Impoverished |
| YERPP | Impoverished |
| YERPS | Impoverished |
| YERPY | Impoverished |
| ACAM1 | Not Enriched |
| ACHLI | Not Enriched |
| ACIAC | Not Enriched |
| ACIAD | Not Enriched |
| ACIB3 | Not Enriched |
| ACIB5 | Not Enriched |
| ACIBC | Not Enriched |
| ACIBS | Not Enriched |
| ACIBT | Not Enriched |
| ACIBY | Not Enriched |
| ACIC1 | Not Enriched |
| ACICJ | Not Enriched |
| ACIF2 | Not Enriched |
| ACIF5 | Not Enriched |
| ACISJ | Not Enriched |
| ACTP2 | Not Enriched |
| ACTP7 | Not Enriched |
| ACTPJ | Not Enriched |
| ACTSZ | Not Enriched |
| AERHH | Not Enriched |
| AERS4 | Not Enriched |
| AGRRK | Not Enriched |
| AGRT5 | Not Enriched |
| ALCBS | Not Enriched |
| ALHEH | Not Enriched |
| ALKMQ | Not Enriched |
| ALKOO | Not Enriched |
| ALTMD | Not Enriched |
| AMOA5 | Not Enriched |
| ANAMM | Not Enriched |
| ANAPZ | Not Enriched |
| ANASP | Not Enriched |
| ANATD | Not Enriched |
| ANAVT | Not Enriched |
| ARTCA | Not Enriched |
| ARTS2 | Not Enriched |
| AYWBP | Not Enriched |
| AZOC5 | Not Enriched |
| AZOPC | Not Enriched |
| AZOSB | Not Enriched |
| AZOSE | Not Enriched |
| BACA2 | Not Enriched |
| BACAH | Not Enriched |
| BACAN | Not Enriched |
| BACC1 | Not Enriched |
| BACC2 | Not Enriched |
| BACC7 | Not Enriched |
| BACCN | Not Enriched |
| BACCR | Not Enriched |
| BACCZ | Not Enriched |
| BACHD | Not Enriched |
| BACHK | Not Enriched |
| BACLD | Not Enriched |
| BACP2 | Not Enriched |
| BACSK | Not Enriched |
| BACSU | Not Enriched |
| BACWK | Not Enriched |
| BEII9 | Not Enriched |
| BIFAA | Not Enriched |
| BIFLD | Not Enriched |
| BIFLO | Not Enriched |
| BORA1 | Not Enriched |
| BORAP | Not Enriched |
| BORBR | Not Enriched |
| BORBU | Not Enriched |
| BORBZ | Not Enriched |
| BORDL | Not Enriched |
| BORGA | Not Enriched |
| BORHD | Not Enriched |
| BORPA | Not Enriched |
| BORPD | Not Enriched |
| BORPE | Not Enriched |
| BORRA | Not Enriched |
| BORT9 | Not Enriched |
| BRAJA | Not Enriched |
| BRASB | Not Enriched |
| BRASO | Not Enriched |
| BREBN | Not Enriched |
| BURP6 | Not Enriched |
| BURP8 | Not Enriched |
| BURPP | Not Enriched |
| BURS3 | Not Enriched |
| BURXL | Not Enriched |
| CALS8 | Not Enriched |
| CAMC1 | Not Enriched |
| CAMC5 | Not Enriched |
| CAMFF | Not Enriched |
| CAMHC | Not Enriched |
| CARHZ | Not Enriched |
| CARRP | Not Enriched |
| CAUCR | Not Enriched |
| CAUSK | Not Enriched |
| CELJU | Not Enriched |
| CHLAB | Not Enriched |
| CHLCH | Not Enriched |
| CHLCV | Not Enriched |
| CHLFF | Not Enriched |
| CHLMU | Not Enriched |
| CHLPD | Not Enriched |
| CHLPN | Not Enriched |
| CHLT2 | Not Enriched |
| CHLTA | Not Enriched |
| CHLTB | Not Enriched |
| CHLTE | Not Enriched |
| CHLTR | Not Enriched |
| CHRSD | Not Enriched |
| CHRVO | Not Enriched |
| CITK8 | Not Enriched |
| CLAM3 | Not Enriched |
| CLAMS | Not Enriched |
| CLOAB | Not Enriched |
| CLOB1 | Not Enriched |
| CLOB8 | Not Enriched |
| CLOBA | Not Enriched |
| CLOBB | Not Enriched |
| CLOBH | Not Enriched |
| CLOBK | Not Enriched |
| CLOBL | Not Enriched |
| CLOBM | Not Enriched |
| CLOCE | Not Enriched |
| CLOD6 | Not Enriched |
| CLOK5 | Not Enriched |
| CLONN | Not Enriched |
| CLOP1 | Not Enriched |
| CLOPE | Not Enriched |
| CLOPH | Not Enriched |
| CLOPS | Not Enriched |
| CLOTH | Not Enriched |
| COLP3 | Not Enriched |
| COPPD | Not Enriched |
| CORDI | Not Enriched |
| COREF | Not Enriched |
| CORGB | Not Enriched |
| CORJK | Not Enriched |
| CORU7 | Not Enriched |
| COXB2 | Not Enriched |
| COXBN | Not Enriched |
| COXBR | Not Enriched |
| COXBU | Not Enriched |
| CYAA5 | Not Enriched |
| CYAP4 | Not Enriched |
| DECAR | Not Enriched |
| DEHE1 | Not Enriched |
| DEHSB | Not Enriched |
| DEHSC | Not Enriched |
| DELAS | Not Enriched |
| DESAP | Not Enriched |
| DESRM | Not Enriched |
| DIAST | Not Enriched |
| DICNV | Not Enriched |
| DICT6 | Not Enriched |
| DICTD | Not Enriched |
| DINSH | Not Enriched |
| ECO24 | Not Enriched |
| ECO27 | Not Enriched |
| ECO7I | Not Enriched |
| ECOHS | Not Enriched |
| ECOK1 | Not Enriched |
| ECOL5 | Not Enriched |
| ECOLC | Not Enriched |
| ECOLU | Not Enriched |
| ECOSM | Not Enriched |
| EHRCJ | Not Enriched |
| EHRCR | Not Enriched |
| EHRRG | Not Enriched |
| EHRRW | Not Enriched |
| ENT38 | Not Enriched |
| ENTFA | Not Enriched |
| ENTS8 | Not Enriched |
| ERWCT | Not Enriched |
| ERWT9 | Not Enriched |
| ERYLH | Not Enriched |
| EXIS2 | Not Enriched |
| FERNB | Not Enriched |
| FINM2 | Not Enriched |
| FRAP2 | Not Enriched |
| FRASC | Not Enriched |
| FRAT1 | Not Enriched |
| FRATF | Not Enriched |
| FRATH | Not Enriched |
| FRATM | Not Enriched |
| FRATN | Not Enriched |
| FRATO | Not Enriched |
| FRATT | Not Enriched |
| FRATW | Not Enriched |
| GEOKA | Not Enriched |
| GEOSL | Not Enriched |
| GEOTN | Not Enriched |
| GLUDA | Not Enriched |
| GLUOX | Not Enriched |
| GRABC | Not Enriched |
| HAEDU | Not Enriched |
| HAEI8 | Not Enriched |
| HAEIE | Not Enriched |
| HAEIG | Not Enriched |
| HAEIN | Not Enriched |
| HAEPS | Not Enriched |
| HAES1 | Not Enriched |
| HAES2 | Not Enriched |
| HAHCH | Not Enriched |
| HALHL | Not Enriched |
| HALOH | Not Enriched |
| HELAH | Not Enriched |
| HELHP | Not Enriched |
| HELMI | Not Enriched |
| HELPG | Not Enriched |
| HELPH | Not Enriched |
| HELPJ | Not Enriched |
| HELPS | Not Enriched |
| HELPY | Not Enriched |
| HYDS0 | Not Enriched |
| HYPNA | Not Enriched |
| IDILO | Not Enriched |
| JANSC | Not Enriched |
| KINRD | Not Enriched |
| KLEP3 | Not Enriched |
| KLEP7 | Not Enriched |
| KOCRD | Not Enriched |
| LACAC | Not Enriched |
| LACBA | Not Enriched |
| LACC3 | Not Enriched |
| LACCB | Not Enriched |
| LACDA | Not Enriched |
| LACDB | Not Enriched |
| LACF3 | Not Enriched |
| LACGA | Not Enriched |
| LACJO | Not Enriched |
| LACLA | Not Enriched |
| LACLM | Not Enriched |
| LACLS | Not Enriched |
| LACPL | Not Enriched |
| LACRD | Not Enriched |
| LACRJ | Not Enriched |
| LACS1 | Not Enriched |
| LACSS | Not Enriched |
| LARHH | Not Enriched |
| LEGPA | Not Enriched |
| LEGPC | Not Enriched |
| LEGPH | Not Enriched |
| LEGPL | Not Enriched |
| LEIXX | Not Enriched |
| LEPCP | Not Enriched |
| LEUCK | Not Enriched |
| LEUMM | Not Enriched |
| LISIN | Not Enriched |
| LISMC | Not Enriched |
| LISMF | Not Enriched |
| LISMH | Not Enriched |
| LISMO | Not Enriched |
| LISW6 | Not Enriched |
| LYSSC | Not Enriched |
| MACCJ | Not Enriched |
| MAGMM | Not Enriched |
| MAGSM | Not Enriched |
| MANSM | Not Enriched |
| MARAV | Not Enriched |
| MARMM | Not Enriched |
| MARMS | Not Enriched |
| MESFL | Not Enriched |
| MESSB | Not Enriched |
| METCA | Not Enriched |
| METFK | Not Enriched |
| METNO | Not Enriched |
| METPB | Not Enriched |
| METPP | Not Enriched |
| METRJ | Not Enriched |
| METS4 | Not Enriched |
| METSB | Not Enriched |
| MICAN | Not Enriched |
| MOOTA | Not Enriched |
| MYCA1 | Not Enriched |
| MYCA5 | Not Enriched |
| MYCAP | Not Enriched |
| MYCBO | Not Enriched |
| MYCBP | Not Enriched |
| MYCCT | Not Enriched |
| MYCGA | Not Enriched |
| MYCGE | Not Enriched |
| MYCGI | Not Enriched |
| MYCH2 | Not Enriched |
| MYCH7 | Not Enriched |
| MYCHJ | Not Enriched |
| MYCLB | Not Enriched |
| MYCLE | Not Enriched |
| MYCMM | Not Enriched |
| MYCMO | Not Enriched |
| MYCMS | Not Enriched |
| MYCPA | Not Enriched |
| MYCPE | Not Enriched |
| MYCPN | Not Enriched |
| MYCPU | Not Enriched |
| MYCS2 | Not Enriched |
| MYCS5 | Not Enriched |
| MYCSJ | Not Enriched |
| MYCSK | Not Enriched |
| MYCSS | Not Enriched |
| MYCTA | Not Enriched |
| MYCTF | Not Enriched |
| MYCTU | Not Enriched |
| MYCUA | Not Enriched |
| MYCVP | Not Enriched |
| NATTJ | Not Enriched |
| NEIG1 | Not Enriched |
| NEIG2 | Not Enriched |
| NEIM0 | Not Enriched |
| NEIMA | Not Enriched |
| NEIMB | Not Enriched |
| NEIMF | Not Enriched |
| NEOSM | Not Enriched |
| NITEC | Not Enriched |
| NITEU | Not Enriched |
| NITMU | Not Enriched |
| NITOC | Not Enriched |
| NOCFA | Not Enriched |
| NOCSJ | Not Enriched |
| NOVAD | Not Enriched |
| OCEIH | Not Enriched |
| OCHA4 | Not Enriched |
| OENOB | Not Enriched |
| ONYPE | Not Enriched |
| ORITB | Not Enriched |
| ORITI | Not Enriched |
| PARDP | Not Enriched |
| PARL1 | Not Enriched |
| PASMU | Not Enriched |
| PEDPA | Not Enriched |
| PELCD | Not Enriched |
| PELPB | Not Enriched |
| PELTS | Not Enriched |
| PHEZH | Not Enriched |
| PHOLL | Not Enriched |
| PHOPR | Not Enriched |
| PHYAS | Not Enriched |
| PHYMT | Not Enriched |
| POLNA | Not Enriched |
| POLSJ | Not Enriched |
| PROM0 | Not Enriched |
| PROM1 | Not Enriched |
| PROM2 | Not Enriched |
| PROM3 | Not Enriched |
| PROM4 | Not Enriched |
| PROM5 | Not Enriched |
| PROM9 | Not Enriched |
| PROMA | Not Enriched |
| PROMH | Not Enriched |
| PROMM | Not Enriched |
| PROMP | Not Enriched |
| PROMS | Not Enriched |
| PROMT | Not Enriched |
| PROVI | Not Enriched |
| PSEA6 | Not Enriched |
| PSEA7 | Not Enriched |
| PSEAB | Not Enriched |
| PSEAE | Not Enriched |
| PSEE4 | Not Enriched |
| PSEF5 | Not Enriched |
| PSEHT | Not Enriched |
| PSEMY | Not Enriched |
| PSEP1 | Not Enriched |
| PSEPF | Not Enriched |
| PSEPG | Not Enriched |
| PSEPK | Not Enriched |
| PSEPW | Not Enriched |
| PSESM | Not Enriched |
| PSEU2 | Not Enriched |
| PSEU5 | Not Enriched |
| PSYA2 | Not Enriched |
| PSYCK | Not Enriched |
| PSYIN | Not Enriched |
| PSYWF | Not Enriched |
| RENSM | Not Enriched |
| RHIE6 | Not Enriched |
| RHIEC | Not Enriched |
| RHIL3 | Not Enriched |
| RHILO | Not Enriched |
| RHILW | Not Enriched |
| RHIME | Not Enriched |
| RHOCS | Not Enriched |
| RHOE4 | Not Enriched |
| RHOFD | Not Enriched |
| RHOP2 | Not Enriched |
| RHOP5 | Not Enriched |
| RHOPB | Not Enriched |
| RHOPT | Not Enriched |
| RHORT | Not Enriched |
| RHOS1 | Not Enriched |
| RHOS4 | Not Enriched |
| RHOS5 | Not Enriched |
| RHOSK | Not Enriched |
| RICAH | Not Enriched |
| RICB8 | Not Enriched |
| RICBR | Not Enriched |
| RICCK | Not Enriched |
| RICCN | Not Enriched |
| RICFE | Not Enriched |
| RICM5 | Not Enriched |
| RICPR | Not Enriched |
| RICRO | Not Enriched |
| RICRS | Not Enriched |
| RICTY | Not Enriched |
| ROSDO | Not Enriched |
| RUTMC | Not Enriched |
| SACD2 | Not Enriched |
| SALA4 | Not Enriched |
| SALAI | Not Enriched |
| SALAR | Not Enriched |
| SALCH | Not Enriched |
| SALDC | Not Enriched |
| SALEP | Not Enriched |
| SALG2 | Not Enriched |
| SALHS | Not Enriched |
| SALNS | Not Enriched |
| SALPA | Not Enriched |
| SALPB | Not Enriched |
| SALPK | Not Enriched |
| SALSV | Not Enriched |
| SALTI | Not Enriched |
| SALTO | Not Enriched |
| SALTY | Not Enriched |
| SERP5 | Not Enriched |
| SHEAM | Not Enriched |
| SHEB2 | Not Enriched |
| SHEB5 | Not Enriched |
| SHEB8 | Not Enriched |
| SHEB9 | Not Enriched |
| SHEDO | Not Enriched |
| SHEFN | Not Enriched |
| SHEHH | Not Enriched |
| SHELP | Not Enriched |
| SHEON | Not Enriched |
| SHEPA | Not Enriched |
| SHEPC | Not Enriched |
| SHEPW | Not Enriched |
| SHESA | Not Enriched |
| SHESH | Not Enriched |
| SHESM | Not Enriched |
| SHESR | Not Enriched |
| SHESW | Not Enriched |
| SHEWM | Not Enriched |
| SHIBS | Not Enriched |
| SHIF8 | Not Enriched |
| SILPO | Not Enriched |
| SILST | Not Enriched |
| SINMW | Not Enriched |
| SPHAL | Not Enriched |
| SPHWW | Not Enriched |
| STAA1 | Not Enriched |
| STAA2 | Not Enriched |
| STAA3 | Not Enriched |
| STAA8 | Not Enriched |
| STAA9 | Not Enriched |
| STAAB | Not Enriched |
| STAAC | Not Enriched |
| STAAE | Not Enriched |
| STAAM | Not Enriched |
| STAAN | Not Enriched |
| STAAR | Not Enriched |
| STAAS | Not Enriched |
| STAAT | Not Enriched |
| STAAW | Not Enriched |
| STAEQ | Not Enriched |
| STAES | Not Enriched |
| STAHJ | Not Enriched |
| STAS1 | Not Enriched |
| STRA1 | Not Enriched |
| STRA3 | Not Enriched |
| STRA5 | Not Enriched |
| STRAW | Not Enriched |
| STRCO | Not Enriched |
| STRE4 | Not Enriched |
| STREM | Not Enriched |
| STRGC | Not Enriched |
| STRGG | Not Enriched |
| STRM5 | Not Enriched |
| STRMK | Not Enriched |
| STRMU | Not Enriched |
| STRP1 | Not Enriched |
| STRP2 | Not Enriched |
| STRP3 | Not Enriched |
| STRP4 | Not Enriched |
| STRP6 | Not Enriched |
| STRP8 | Not Enriched |
| STRPB | Not Enriched |
| STRPC | Not Enriched |
| STRPD | Not Enriched |
| STRPF | Not Enriched |
| STRPG | Not Enriched |
| STRPI | Not Enriched |
| STRPM | Not Enriched |
| STRPN | Not Enriched |
| STRPS | Not Enriched |
| STRPZ | Not Enriched |
| STRR6 | Not Enriched |
| STRS2 | Not Enriched |
| STRS7 | Not Enriched |
| STRSV | Not Enriched |
| STRSY | Not Enriched |
| STRT2 | Not Enriched |
| STRTD | Not Enriched |
| STRZJ | Not Enriched |
| STRZP | Not Enriched |
| SULMW | Not Enriched |
| SULNB | Not Enriched |
| SULSY | Not Enriched |
| SYMTH | Not Enriched |
| SYNJA | Not Enriched |
| SYNP2 | Not Enriched |
| SYNP6 | Not Enriched |
| SYNPW | Not Enriched |
| SYNPX | Not Enriched |
| SYNR3 | Not Enriched |
| SYNS3 | Not Enriched |
| SYNS9 | Not Enriched |
| SYNSC | Not Enriched |
| SYNWW | Not Enriched |
| SYNY3 | Not Enriched |
| THEAB | Not Enriched |
| THEEB | Not Enriched |
| THEFY | Not Enriched |
| THEM4 | Not Enriched |
| THEP1 | Not Enriched |
| THEP3 | Not Enriched |
| THEPX | Not Enriched |
| THETN | Not Enriched |
| THICR | Not Enriched |
| THIDA | Not Enriched |
| THISH | Not Enriched |
| TREPA | Not Enriched |
| TREPS | Not Enriched |
| TRIEI | Not Enriched |
| TROW8 | Not Enriched |
| TROWT | Not Enriched |
| UNCTG | Not Enriched |
| UREP2 | Not Enriched |
| UREPA | Not Enriched |
| UREU1 | Not Enriched |
| VEREI | Not Enriched |
| VESOH | Not Enriched |
| VIBC3 | Not Enriched |
| VIBCH | Not Enriched |
| VIBCM | Not Enriched |
| VIBF1 | Not Enriched |
| VIBFM | Not Enriched |
| VIBHB | Not Enriched |
| VIBPA | Not Enriched |
| VIBSL | Not Enriched |
| VIBVU | Not Enriched |
| VIBVY | Not Enriched |
| WOLPM | Not Enriched |
| WOLPP | Not Enriched |
| WOLTR | Not Enriched |
| WOLWR | Not Enriched |
| XANAC | Not Enriched |
| XANC5 | Not Enriched |
| XANC8 | Not Enriched |
| XANCB | Not Enriched |
| XANCP | Not Enriched |
| XANOM | Not Enriched |
| XANOP | Not Enriched |
| XANOR | Not Enriched |
| XANP2 | Not Enriched |
| XYLF2 | Not Enriched |
| XYLFA | Not Enriched |
| XYLFM | Not Enriched |
| XYLFT | Not Enriched |
| ZYMMO | Not Enriched |
| ACIBL | Over Enriched |
| ACIC5 | Over Enriched |
| AKKM8 | Over Enriched |
| ANADE | Over Enriched |
| ANADF | Over Enriched |
| ANASK | Over Enriched |
| AQUAE | Over Enriched |
| ARCB4 | Over Enriched |
| ARTAT | Over Enriched |
| BACFN | Over Enriched |
| BACFR | Over Enriched |
| BACTN | Over Enriched |
| BACV8 | Over Enriched |
| BDEBA | Over Enriched |
| CAMJ8 | Over Enriched |
| CAMJD | Over Enriched |
| CAMJE | Over Enriched |
| CAMJJ | Over Enriched |
| CAMJR | Over Enriched |
| CAMLR | Over Enriched |
| CHLAA | Over Enriched |
| CHLAD | Over Enriched |
| CHLL2 | Over Enriched |
| CHLP8 | Over Enriched |
| CHLPB | Over Enriched |
| CHLSY | Over Enriched |
| CHLT3 | Over Enriched |
| CLOTE | Over Enriched |
| CORGL | Over Enriched |
| CYTH3 | Over Enriched |
| DEIDV | Over Enriched |
| DEIGD | Over Enriched |
| DEIRA | Over Enriched |
| DESAA | Over Enriched |
| DESDG | Over Enriched |
| DESHY | Over Enriched |
| DESOH | Over Enriched |
| DESPS | Over Enriched |
| DESVH | Over Enriched |
| DESVV | Over Enriched |
| ELUMP | Over Enriched |
| FLAJ1 | Over Enriched |
| FLAPJ | Over Enriched |
| FRAAA | Over Enriched |
| FRASN | Over Enriched |
| FUSNN | Over Enriched |
| GEOBB | Over Enriched |
| GEOLS | Over Enriched |
| GEOMG | Over Enriched |
| GEOUR | Over Enriched |
| GLOVI | Over Enriched |
| GRAFK | Over Enriched |
| HERA2 | Over Enriched |
| LAWIP | Over Enriched |
| LEPBA | Over Enriched |
| LEPBJ | Over Enriched |
| LEPBL | Over Enriched |
| LEPBP | Over Enriched |
| LEPIC | Over Enriched |
| LEPIN | Over Enriched |
| METI4 | Over Enriched |
| MYCA9 | Over Enriched |
| MYXXD | Over Enriched |
| NAUPA | Over Enriched |
| NITSB | Over Enriched |
| NOSP7 | Over Enriched |
| OPITP | Over Enriched |
| PARD8 | Over Enriched |
| PARUW | Over Enriched |
| PELLD | Over Enriched |
| PELPD | Over Enriched |
| PELUB | Over Enriched |
| PETMO | Over Enriched |
| PORG3 | Over Enriched |
| PORGI | Over Enriched |
| PROA2 | Over Enriched |
| PROAC | Over Enriched |
| RHOBA | Over Enriched |
| RHOOB | Over Enriched |
| RHOSR | Over Enriched |
| ROSCS | Over Enriched |
| ROSS1 | Over Enriched |
| RUBXD | Over Enriched |
| SACEN | Over Enriched |
| SALRD | Over Enriched |
| SOLUE | Over Enriched |
| SORC5 | Over Enriched |
| SULDN | Over Enriched |
| SYNAS | Over Enriched |
| SYNFM | Over Enriched |
| SYNJB | Over Enriched |
| THELT | Over Enriched |
| THEMA | Over Enriched |
| THERP | Over Enriched |
| THESQ | Over Enriched |
| THET2 | Over Enriched |
| THET8 | Over Enriched |
| TREDE | Over Enriched |
| WOLSU | Over Enriched |
