# Supplementary material for: Proteome-Wide Analysis of Functional Divergence in Bacteria: Exploring a Host of Ecological Adaptations
Source: PLoS One. 2012 Apr 26;7(4):e35659. doi: 10.1371/journal.pone.0035659 (PMC3338524; doi:10.1371/journal.pone.0035659)
Supplement: Table S3 — Effect of bacterial lifestyle and genome size on functional divergence. We used a generalized linear model with binomial errors to assess the impact of lifestyle and genome size on the enrichment and impoverishment of genomes for functional divergence. The saturated model was fit with the glm function in R, and simplified to a minimal adequate model with the step function, which determined that the interaction was not significant. Both lifestyle and genome size have a significant impact on enrichment status, with host-associated bacteria and bacteria with larger genomes more likely to be impoverished for functional divergence. (DOCX) [file pone.0035659.s004.docx]

| **Term** | **Coefficient** | **P** |
| --- | --- | --- |
| Intercept | 2.265 | 8.23 x 10^-7^ |
| Lifestyle (Host-assoc.) | -2.536 | 1.28 x 10^-13^ |
| Genome size (bp.) | -1.799 x 10^-7^ | 0.0212 |
