# Supplementary material for: Proteome-Wide Analysis of Functional Divergence in Bacteria: Exploring a Host of Ecological Adaptations
Source: PLoS One. 2012 Apr 26;7(4):e35659. doi: 10.1371/journal.pone.0035659 (PMC3338524; doi:10.1371/journal.pone.0035659)
Supplement: Table S4 — Enrichment status of gene categories in host-associated and free-living bacteria. Categories U and T show different levels of enrichment for functional divergence when the analysis is run on these groups of bacteria independently. (DOCX) [file pone.0035659.s005.docx]

| COG category | Free-living | Host-associated |
| --- | --- | --- |
| C | Enriched | Enriched |
| P | Enriched | Enriched |
| M | Enriched | Enriched |
| V | Enriched | Enriched |
| L | Impoverished | Impoverished |
| D | Impoverished | Impoverished |
| Q | Enriched | Enriched |
| G | Enriched | Enriched |
| E | Enriched | Enriched |
| J | Impoverished | Impoverished |
| U | Not Enriched | Enriched |
| T | Enriched | Impoverished |
| K | Impoverished | Impoverished |
| O | Enriched | Enriched |
| N | Not Enriched | Not Enriched |
| I | Impoverished | Impoverished |
| H | Enriched | Enriched |
| F | Enriched | Enriched |
| A | Impoverished | Impoverished |
