# Supplementary material for: Proteome-Wide Analysis of Functional Divergence in Bacteria: Exploring a Host of Ecological Adaptations
Source: PLoS One. 2012 Apr 26;7(4):e35659. doi: 10.1371/journal.pone.0035659 (PMC3338524; doi:10.1371/journal.pone.0035659)
Supplement: Table S5 — Sites under functional divergence in VirB8. The left column shows the sites found in E.coli UMN026, the sites on the right show the homologous sites in Agrobacterium tumefaciens. Sites without a value for Agrobacterium tumefaciens represent sites, which have not been crystalised. (DOCX) [file pone.0035659.s006.docx]

| *E. coli* UMN026 | *Agrobacterium tumefaciens* |
| --- | --- |
| R11 |  |
| D21 |  |
| T25 |  |
| V57 |  |
| D58 |  |
| Y60 |  |
| D63 |  |
| H71 |  |
| T73 |  |
| Y116 | F127 |
| L176 | V183 |
| D189 | T196 |
| E206 | L211 |
| T213 | V218 |
