# Supplementary material for: Proteome-Wide Analysis of Functional Divergence in Bacteria: Exploring a Host of Ecological Adaptations
Source: PLoS One. 2012 Apr 26;7(4):e35659. doi: 10.1371/journal.pone.0035659 (PMC3338524; doi:10.1371/journal.pone.0035659)
Supplement: Table S6 — Detecting functional divergence using BioNJ trees. The number of FD sites predicted at the 0.05 p-value level using BioNJ trees is consistently smaller. This indicates a stricter scoring scheme, which potentially reduces false positives. It can be seen from the last column, that the percentage of the FD sites detected through BioNJ trees that are also detected through ML trees increases with larger number of sequences in the alignment and approaches 100%. Only the smaller alignments show noticeable discrepancies but some of these can already be explained by the effect of different tree topologies. Overall, the above results confirm the suitability of BioNJ for tree construction, particularly for alignments with a large number of sequences. (DOCX) [file pone.0035659.s007.docx]

|  | Number of FD sites ML tree | Number of FD sites BIONJ tree | Overlapping sites | Percentage of BioNJ contained in ML |
| --- | --- | --- | --- | --- |
| 26 | 10 | 3 | 3 | 100% |
| 34 | 29 | 8 | 3 | 37.5% |
| 83 | 72 | 40 | 23 | 57.5% |
| 204 | 256 | 102 | 88 | 86% |
| 348 | 134 | 87 | 82 | 94% |
| 692 | 298 | 226 | 219 | 97% |
| 881 | 293 | 230 | 225 | 98% |
|  |  |  |  |  |
| Total | 1092 | 696 | 643 | 92% |
