# Supplementary material for: Proteome-Wide Analysis of Functional Divergence in Bacteria: Exploring a Host of Ecological Adaptations
Source: PLoS One. 2012 Apr 26;7(4):e35659. doi: 10.1371/journal.pone.0035659 (PMC3338524; doi:10.1371/journal.pone.0035659)
Supplement: Table S7 — Comparison of runtime for two methods of functional divergence. The most widely used program for detection of functional divergence is DIVERGE [81]. Even though it is well-suited for individual analyses, it can not be used for a large-scale study such as the one presented here. This is because the size of alignments dealt with exceeds the limits of the data that DIVERGE can handle. It is also not designed to be run automatically. (DOCX) [file pone.0035659.s008.docx]

| #Species/Sequences | Alingment Length in amino acids | Diverge Time(Gu99) NJ tree | CAFS with calculated BioNJ tree |
| --- | --- | --- | --- |
| 10 | 334 | NTN | NTN |
| 26 | 510 | 11s | 26s |
| 34 | 247 | 10s | 1m30s |
| 86 | 316 | 3m16s | 2m57s |
| 204 | 492 | Failed* | 47m21s |
| 348 | 550 | Failed** | 2h21m12s |
| 692 | 587 | Failed* | 9h21m28s |
| 881 | 1101 | Failed* | 20h20m54s |
|  |  |  |  |
