# Supplementary material for: Proteome-Wide Analysis of Functional Divergence in Bacteria: Exploring a Host of Ecological Adaptations
Source: PLoS One. 2012 Apr 26;7(4):e35659. doi: 10.1371/journal.pone.0035659 (PMC3338524; doi:10.1371/journal.pone.0035659)
Supplement: Table S8 — Functionalities of CAFS in comparison with DIVERGE. (DOCX) [file pone.0035659.s009.docx]

|  | **DIVERGE** | **OUR SOFTWARE** |
| --- | --- | --- |
| Tree building method | NJ with Poison | **BioNJ with JTT** |
| Can accept prebuilt tree | **Yes** | **Yes** |
| Can automate large analyses | No | **Yes** |
| Can test every node | No | **Yes** |
| Largest Alignment Readable | 100species x 316AA | **3179species x 8050AA**  (So far) |
| Open Source Code | No | **Yes** |
| Can use tagging system for clustering analysis | No | **Yes** |
| Gui | **Yes** | No |
| Test Statistic | **Bayesian based** | Distance based |
| Simulations to assess significance | No | **Yes** |
| Operating Systems | Windows | **Mac and Linux with Windows availability via cygwin.** |
