# Supplementary material for: Proteome-Wide Analysis of Functional Divergence in Bacteria: Exploring a Host of Ecological Adaptations
Source: PLoS One. 2012 Apr 26;7(4):e35659. doi: 10.1371/journal.pone.0035659 (PMC3338524; doi:10.1371/journal.pone.0035659)
Supplement: Text S1 — Justification for use of BioNJ and Comparison of results performed with Maximum Likelihood trees vs BIONJ trees. (DOCX) [file pone.0035659.s010.docx]

**Justification for use of BioNJ**

There are two main reasons why the use of BioNJ trees was chosen over maximum likelihood trees in this study. First of all, it runs much faster, which is critical for large-scale applications. Secondly, we feel that incorporating a maximum likelihood calculation (RaxML or PAML for example) would have a negative impact on both use by the less computer savy users as they would need an in depth knowledge of the ML package and would also mean that future versions of this software would be constrained by the ML package. We feel that these things would affect the automation of large scale analyses and also the out “of the box” feel of the program. Additionally the maximum likelihood methods employed in the Bio++ libraries which we use in our program are significantly slower than RaxML.

**Comparison of results performed with Maximum Likelihood trees vs BIONJ trees**

Table S6 details the extra time that it takes to construct Maximum Likelihood (ML) trees prior to running our software. The trees were generated using RaxML-Pthreads on a 4-core machine for a selection of eight alignments. These eight alignments were chosen as representatives for bins of various input sizes. Different tree topologies lead to minor differences in the number of testable nodes between the two methods. The extrapolated time is an estimate of the time it would take to obtain a complete set of ML trees for the dataset used in this study. In this case the overhead for ML tree construction on a 4-core computer would amount to more than a year of computation. Greater differences in the smaller alignments can be accounted for by the test statistic. This test statistic (equation 1 in the main manuscript) depends on two characteristics, firstly the numerator will be large (either positive or negative) for a column in an alignment if there is a large amount of conservation in each clade whilst the amino acids in each clade are radically different. Secondly the denominator depends on the variance in the aforementioned clades. These two factors mean that smaller alignments are subject to larger errors.

To check for the quality of results from BioNJ trees compared to ML trees we investigated the overlap between the FD sites from each method, as shown in Table S2.
