# Supplementary material for: Proteome-Wide Analysis of Functional Divergence in Bacteria: Exploring a Host of Ecological Adaptations
Source: PLoS One. 2012 Apr 26;7(4):e35659. doi: 10.1371/journal.pone.0035659 (PMC3338524; doi:10.1371/journal.pone.0035659)
Supplement: Text S2 — A Note about testable alignments and computation time for functional divergence detection. It should be noted that since DIVERGE could not run the 4 largest alignments in this subset of our dataset, we predict that at least half of the alignments in our full dataset can not be analysed by the DIVERGE software. Indeed we were unable to run analysis on any alignment over 86 sequences long, however DIVERGE would read alignments up to 100 sequences long. Another further problem about the calculations performed with DIVERGE2.0 is the impossibility to perform analyses collected in Gu2001 which pertains to the method in [81]. This analysis did not work for any of the alignments above. All alignments failed with the error “Please recheck input sequence data and tree information”, for which we could not find documentation. In addition to the times stated above we would like to add that DIVERGE works on an alignment by alignment basis and as such the user must manually chose files to be analysed and also the trees to be input and the nodes to be tested. This makes proteome level analyses prohibitive. The runtimes given in the table above do not include the time it takes for the user to choose nodes for functional divergence testing with DIVERGE. (DOCX) [file pone.0035659.s011.docx]

It should be noted that since DIVERGE could not run the 4 largest alignments in this subset of our dataset, we predict that at least half of the alignments in our full dataset can not be analysed by the DIVERGE software. Indeed we were unable to run analysis on any alignment over 86 sequences long, however DIVERGE would read alignments up to 100 sequences long. Another further problem about the calculations performed with DIVERGE2.0 is the impossibility to perform analyses collected in Gu2001 which pertains to the method in (Gu and Vander Velden 2002). This analysis did not work for any of the alignments above. All alignments failed with the error “Please recheck input sequence data and tree information”, for which we could not find documentation.

In addition to the times stated above we would like to add that DIVERGE works on an alignment by alignment basis and as such the user must manually chose files to be analysed and also the trees to be input and the nodes to be tested. This makes proteome level analyses prohibitive. The runtimes given in the table above do not include the time it takes for the user to choose nodes for functional divergence testing with DIVERGE.
