# Supplementary material for: Proteome-Wide Analysis of Functional Divergence in Bacteria: Exploring a Host of Ecological Adaptations
Source: PLoS One. 2012 Apr 26;7(4):e35659. doi: 10.1371/journal.pone.0035659 (PMC3338524; doi:10.1371/journal.pone.0035659)
Supplement: Text S3 — Conclusion of Comparisons of the methods to identify functional divergence. We conclude that whilst we understand and acknowledge the value of building maximum likelihood trees we feel that one of the biggest assetts of our program is speed and automation. With this in mind, we wish to demonstrate to the user that whilst BioNJ trees are calculated within the program the results recieved are ∼92% comparable to those returned after the maximum likelihood analysis. Given the high level of similarity between the maximum likelihood built trees and those built with BioNJ we feel that the conclusions drawn in the manuscript would hold in either circumstance. We also feel that analyses on the scale of those detailed in the main text are not possible for many who have limited computational resources. In reference to the DIVERGE comparisons we feel justified in our calculations that DIVERGE is not ideal for large scale analyses and can be troublesome to the user for even relatively small alignments. In addition to the tests run above we would like to say that on large scale analyses all of the information about the sites tested are collected and automatically analysed according to any tagging system applied to the dataset. (DOCX) [file pone.0035659.s012.docx]

We conclude that whilst we understand and acknowledge the value of building maximum likelihood trees we feel that one of the biggest assetts of our program is speed and automation. With this in mind, we wish to demonstrate to the user that whilst BioNJ trees are calculated within the program the results recieved are ~92% comparable to those returned after the maximum likelihood analysis. Given the high level of similarity between the maximum likelihood built trees and those built with BioNJ we feel that the conclusions drawn in the manuscript would hold in either circumstance. We also feel that analyses on the scale of those detailed in the main text are not possible for many who have limited computational resources.

In reference to the DIVERGE comparisons we feel justified in our calculations that DIVERGE is not ideal for large scale analyses and can be troublesome to the user for even relatively small alignments.

In addition to the tests run above we would like to say that on large scale analyses all of the information about the sites tested are collected and automatically analysed according to any tagging system applied to the dataset.

**References**

**Gu, X. and K. Vander Velden (2002). "DIVERGE: phylogeny-based analysis for functional-structural divergence of a protein family." Bioinformatics 18(3): 500-501.**
